# Vitamin D Receptor Polymorphisms in Sex-Frailty Paradox

**DOI:** 10.3390/nu12092714

**Published:** 2020-09-05

**Authors:** Beatrice Arosio, Franca Rosa Guerini, Andrea Saul Costa, Alessandra Dicitore, Evelyn Ferri, Daniela Mari, Erminio Torresani, Mario Clerici, Matteo Cesari, Giovanni Vitale

**Affiliations:** 1 Geriatric Unit, Fondazione IRCCS Ca’ Granda Ospedale Maggiore Policlinico, 20122 Milan, Italy; beatrice.arosio@unimi.it (B.A.); evelyn.ferri@guest.unimi.it (E.F.); matteo.cesari@unimi.it (M.C.); 2 Department of Clinical Sciences and Community Health, University of Milan, 20122 Milan, Italy; giovanni.vitale@unimi.it; 3 IRCCS Fondazione Don Carlo Gnocchi, 20148 Milan, Italy; acosta@dongnocchi.it (A.S.C.); mario.clerici@unimi.it (M.C.); 4 Laboratorio Sperimentale di Ricerche di Neuroendocrinologia Geriatrica ed Oncologica, Istituto Auxologico Italiano, IRCCS, 20095 Cusano Milanino, Italy; alessandra.dicitore@libero.it (A.D.); daniela.mari@unimi.it (D.M.); 5 Laboratorio Analisi Cliniche Centro di Ricerche e Tecnologie Biomediche, Istituto Auxologico Italiano, IRCCS, 20095 Cusano Milanino, Italy; e.torresani@auxologico.it; 6 Department of Pathophysiology and Transplantation, University of Milan, 20122 Milan, Italy

**Keywords:** aging, vitamin D, frailty, *vitamin D receptor*

## Abstract

The “male-female health-survival paradox” evidences that the survival advantage observed in women is linked to higher rates of disability and poor health status compared to men, a phenomenon also called the “sex-frailty paradox”. The depletion of vitamin D seems to play a role in the fragilization of old persons, and genetic polymorphisms of the *vitamin D receptor* (*VDR*) gene seem to be involved in regulating the vitamin D pathway. This study correlated the *VDR* gene polymorphisms (FokI, ApaI, BsmiI, and TaqI) with frailty, computed by frailty index (FI), in 202 persons (127 women and 75 men, aged from 60 to 116 years), aiming to capture the involvement of vitamin D in the sex-frailty paradox. The results showed slightly higher FI (*p* = 0.05), lower levels of 25(OH)D (*p* = 0.04), and higher levels of parathyroid hormone PTH (*p* = 0.002) and phosphorus (*p* < 0.001) in women than in men. Interestingly, the ApaI minor allele (Aa + aa) showed a significant positive association with FI (*p* = 0.03) and a negative association with inorganic phosphorus values (*p* = 0.04) compared to AA genotype only in women, regardless of age. The exact mechanism and the causal role that, in old women, links ApaI polymorphism with frailty are still unclear. However, we could speculate that a specific genetic profiling, other than 25(OH)D levels, play a role in the sex-frailty paradox.

## 1. Introduction

The worldwide increase of human life expectancy and the concomitant rapid aging of the population represent major demographic phenomena of the last century.

It has been known that aging has different effects in women and men: a phenomenon described as the “male-female health-survival paradox”, also known as the “sex-frailty paradox” [1], in which women experience greater longevity than men [2]. However, this survival advantage in women is linked to higher rates of disability and poor health during their lives, and characterized by more chronic diseases compared to men [1].

The higher clinical complexity is well represented by the concept of frailty that is a typical condition in older persons. It is characterized by increased vulnerability to stressors and reduced homeostatic reserves [3].

Such risk conditions seem to differently impact on women and men. Indeed, the sex-frailty paradox has recently been explained with the proposal that women may experience a different kind of frailty: though they manifest a poorer health status compared to men, such higher vulnerability does not translate into a higher risk of death [4,5,6].

Moreover, epigenetic changes, environmental factors, and anthropological culture that differently affect men and women’s lives may play a crucial role and, together with the genetic profile, concur at the definition of the sex-frailty paradox [7].

Recent studies have identified the depletion of vitamin D as a key factor contributing to the development of frailty in very old persons [8]. Strategies to prevent hypovitaminosis D have been proposed as useful to slow down the processes of “fragilization” naturally occurring during aging [9,10].

The action of this vitamin is mediated by the vitamin D receptor (VDR), a nuclear transcription-regulating factor that drives the synthesis of proteins involved in bone mineral homeostasis and cell cycle regulation. The (1,25(OH)_2_D)-VDR complex is transferred to the nucleus where it generates heterodimers with the retinoid X receptor (RXR), which binds a specific DNA sequence called vitamin D response element (VDRE). This results in the activation of a plethora of genes.

The *VDR* gene is located on the chromosome 12.13.11. It encompasses different single nucleotide polymorphisms (SNPs), including ApaI (rs7975232) [11] and BsmI (rs1544410) [12], located in intron 8, TaqI (rs731236) in exon 9, and [13] the missense FokI (rs2228570) in exon 2.

Interestingly, in a cohort study of centenarians we have previously identified an association between these SNPs with functional, physical, and cognitive performances [14]. Consistently, in this cohort, certain genetic profiles were related to different prevalence of age-related diseases, such as hypertension, acute myocardial infarction, angina, venous insufficiency, dementia, chronic obstructive pulmonary disease, and arthrosis [14].

Starting from these premises, we analyzed possible associations between frailty and the vitamin D pathway as well as *VDR* gene profiling in a cohort of very old women and men. In this study, we used the frailty index (FI) as measure of frailty because it is designed to estimate the biological aging of the individual through a quantitative (i.e., the age-related accumulation of health deficits) [15] and multidimensional [16] approach.

## 2. Materials and Methods

### 2.1. Study Design

Data are from a cohort study conducted in Northern Italy between 2007 and 2014 and funded by the Italian Ministry of University and Scientific Research.

A trained multidisciplinary team administered a standardized and structured questionnaire to all subjects to record information about their health, functions, medications use, clinical history, and lifestyle. In particular, the research personnel went to each centenarian’s house or nursing home to administer the evaluation.

A total of 202 persons (127 women and 75 men) aged between 60 and 116 years old were enrolled. These participants did not take any medications affecting vitamin D pathway.

The study protocol received approval from the local ethical committee. All subjects who gave written informed consent to participate in the study and filled out the questionnaire, were included in the study.

### 2.2. Frailty Index

A FI was computed following the criteria described by Searle et al. [17] and taking into account a wide range of age-related signs, symptoms, disabilities, and diseases.

Briefly, the constituting variables were scored as 0 (absence of the deficit) or 1 (presence of the deficit). The FI was then calculated as the ratio between the number of health deficits presented by the individual and the total number of health deficits considered for its computation (in our case, n = 30), as previously described [16].

### 2.3. Biochemical Analyses

Serum parathyroid hormone PTH and 25(OH) vitamin D concentrations were measured through Cobas electrochemiluminescence immunoassays (ECLIA) on the Modular E170 analyzer (Roche Diagnostics, Mannheim, Germany). Serum calcium and phosphate (inorganic) were measured with Cobas^®^ CA2 and Cobas^®^PHOS2 on COBAS C702 analyzer (Roche Diagnostics, Mannheim, Germany), employing as test principle 5-nitro-5′-methyl-BAPTA (NM-BAPTA) and molybdate UV reactions, respectively. All assays were performed according to the recommendations of the respective manufacturers.

The range of vitamin D are: >30 μg/L normal, 21–29 μg/L insufficient, and <20 μg/L lacking levels. The coefficient of variation ranges from 4.8% to 10.8% (mean values from 15.0 to 27.7). For the other parameters, the normal range values are: PTH 13–64 ng/L, calcium 8.1–10.4 mg/dL and inorganic phosphorus 2.5–4.5 mg/dL.

### 2.4. VDR Genotyping

For the genetic analyses, 85 out of 202 subjects were analyzed (117 subjects did not provide informed consent for these analyses). The following *VDR* polymorphisms were analyzed: rs731236 A/G(T/t) (TaqI), rs10735810 C/T(F/f) (FokI), rs1544410 C/T(B/b) (BsmI), and rs7975232 A/C(A/a) (ApaI). DNA was isolated from peripheral blood through the phenol/chloroform method.

SNPs were evaluated by Allelic Discrimination Real-time PCR using pre-designed TaqMan probes (Applied Biosystems, Foster City, CA). PCR consisted of a hot start at 95 °C for 10 min followed by 40 cycles of 94 °C for 15 sec and 60 °C for 1 min. Fluorescence detection took place at 60 °C. Assays were performed in 10 μL reactions, using TaqMan Genotyping Master Mix on 96-well plates using an ABI 7000 instrument (Applied Biosystems). Control samples, representing all possible genotypes and a negative control, were included in each reaction.

### 2.5. Statistical Analysis

Chi-square analysis was used to verify that populations were in Hardy–Weinberg Equilibrium (HWE) and to evaluate the sex association. Linear regression analysis was adopted to evaluate the FI association with the *VDR* genotype distribution in men and women, adjusting by age. Multivariate stepwise binary logistic regression analysis was performed to evaluate the association between *VDR* genotype and biochemical parameters. Evaluation was conducted separately in men and women and adjusted by age and FI. Analyses were carried out using SPSS 26.0 for Windows.

Linkage disequilibrium and haplotype gender association were calculated by SHEsis [18] and haplotype correlation with FI as well as with biochemical parameters was calculated by regression analysis, adjusting by age, using PLINK software [19].

## 3. Results

### 3.1. FI and Vitamin D Pathway in Women and Men

Table 1 reports the main characteristics of the study sample according to sex. No difference was observed in the mean age of women and men. The FI was slightly higher in women than in men (*p* = 0.05). Conversely, significantly lower levels of 25(OH)D (*p* = 0.04) and higher levels of PTH and phosphorus were evidenced in women compared to men (*p* = 0.002 and *p* < 0.001, respectively). No significant difference was found for calcium levels.

As expected, due to the advanced age of the participants, all subjects were 25(OH)D-deficient and the women showed higher PTH levels than normal ranges.

### 3.2. VDR Association with FI and Vitamin D Pathway

The *VDR* rs73236 (ApaI), rs7975232 (TaqI), rs10735810 (FokI), and rs1544410 (BsmI) analyses were performed. Genotype distributions evidenced a low skewing of ApaI frequency from HWE equilibrium in women (*p* = 0.02). In particular, a higher frequency of ApaI Aa genotype was present in women than expected. No different distributions of the *VDR* gene polymorphisms were observed between women and men (Table S1).

In a subsequent analysis, we clustered the subjects into carriers of the minor allele (i.e., heterozygous + homozygous for minor): TaqI (Tt + tt), BsmI (Bb + bb), ApaI (Aa + aa), and FokI (Ff + ff) and carriers of the homozygote major allele: TaqI (TT), BsmI (BB), ApaI (AA), and FokI (FF).

Women carrying the minor ApaI allele (Aa + aa) showed significantly higher FI values (*p* = 0.03) (Table 2). No association was observed in men.

The multivariate stepwise binary logistic analysis evidenced in women a significant association between ApaI (Aa + aa) genotype with low levels of inorganic phosphorus (*p* = 0.04 OR = 0.18 95% IC: 0.03–0.9). No association was observed between ApaI polymorphism and 25(OH)D (*p* = 0.10) nor with PTH (*p* = 0.42). Independently of the genetic profile, phosphorus correlated with FI only in women (R = 0.21 *p* = 0.03), but not in men.

Linkage haplotype study was conducted separately in women and men to evaluate the linkage score between *VDR* variants as well as to verify the presence of association between the different haplotypes and sex. Haplotype analysis evidenced, as expected, the presence of a linkage disequilibrium (LD) between ApaI, BsmI, and TaqI (r2 > 0.3); in contrast, no linkage was detected between FokI and any of the other *VDR* polymorphisms (r2 < 0.10). Haplotype distributions were not significantly different between men and women.

## 4. Discussion

The main finding of this study was the association between *VDR* ApaI gene polymorphism and FI values in very old women. Interestingly, we have previously described the impact of *VDR* SNPs on different age-related diseases comparing the subjects by age and not by sex [14]. To our knowledge, this is the first study that correlates *VDR* ApaI SNP with frailty by the means of the FI computation.

How to characterize a person’s health status is not clear. Multidimensional approaches that aggregate the individual effects of multiple biological and physiological markers into an aging score have already been studied. The FI, based on the assumption that health deficits tend to accumulate with aging, represents a quantitative measure of extreme interest which is able to capture the complexity of the aging phenomenon [20].

It has been known that aging has different effects in women and men. The sex-frailty paradox is a well-described phenomenon, in which women have experienced greater longevity than men since the 18^th^ century. However, this survival advantage in females is linked to higher rates of disability and poor health status during their lives, as demonstrated by the higher FI shown by women compared men in this cohort.

The remodeling of the endocrine system could play a key role in the different aging trajectories [21,22] and may explain the different rate of survival between the sexes [23]. In particular, this remodeling involves the parathyroid glands altering the production of PTH and, consequently, the entire vitamin D pathway [24]. In general, the consequences of these pathway changes are manifold and include the reduction of bone and skeletal muscle mass, the reduction in muscle strength, and the increase in adipose tissue. Interestingly, significant correlation was found between serum levels of vitamin D and muscle strength/physical performance mainly in women [25].

All these modifications can influence and drive the “fragilization” of the older person [8,26], particularly in women. In addition, vitamin D appears to have a role in the development of several age-related diseases (cardiovascular diseases, diabetes, cancer, etc.) [27].

Due to the older age of our subjects, the levels of vitamin D were lower than normal ranges in both women and men. Interestingly, we observed significantly different levels not only of 25(OH)D, but also of PTH and inorganic phosphorus in women compared to men. This result may be a consequence of the 25(OH)D and PTH hormonal axis imbalance in regulating phosphorus levels, especially in women [28,29,30,31]. Conversely, no differences were shown for calcium. In women, low levels of 25(OH)D seem to be related to frailty differently than men, in accordance with the literature [32].

Most of the biological activities of 25(OH)D are mediated by VDR, a high-affinity receptor acting as a ligand-activated transcription factor. Generally, the age-related reduction of the VDR expression seems to be more evident in women than in men [33]. Alterations of the *VDR* gene have been hypothesized to cause important defects in gene activation, affecting calcium metabolism, cell proliferation, immune function, PTH synthesis, and intestinal absorption of calcium and phosphate [34]. As a matter of fact, *VDR* polymorphisms have been largely associated with different diseases as well as with successful aging [14].

Interestingly, in women carrying the minor allele of the ApaI polymorphism, we found higher FI values and lower phosphorus levels compared to women homozygous for the major allele, independently of age.

It is of note that the metabolism of phosphorus is regulated by PTH, 25(OH)D and fibroblast growth factor 23 (FGF23) and, in turn, VDR regulates the gene expression of FGF23 as well as Klotho [35]. Klotho is a bona fide longevity gene that, if inactivated, generates a phenotype identical to that of FGF23-null mice [36], characterized by premature aging, decreased lifespan, elevated 25(OH)D, hyperphosphatemia, ectopic calcification, skin and muscle atrophy, osteoporosis, and hearing loss.

Although the functional role of the VDR polymorphisms is not well defined, it has been reported that ApaI together with BsmI and TaqI cause silent codon changes influencing the different stability and translation of the *VDR* mRNA [33] and, probably, the VDR levels.

Interestingly, in patients with chronic renal failure, the *VDR* genetic profile seems to perturb the FGF23 expression and consequently the physiological action of FGF23 mediated by FGF receptor and co-receptor Klotho [37].

Since ApaI minor allele per se or in linkage with BsmI is associated with different diseases such as Alzheimer’s disease [38], osteoarthritis, rheumatoid arthritis [39], and osteopenia [40], we could speculate that ApaI minor allele is related to the “fragilization” of old women, playing a role in the low 25(OH)D levels in the sex-frailty paradox. The exact mechanism involved and whether there is a causal role is still unclear. We can postulate that a specific *VDR* genetic profile may impact on phosphorus levels in old women, modulating genes involved in aging [41].

This is the first study that underlines an association between a specific genetic profile of the *VDR* gene and frailty.

The limitations of our study may be the low number of men compared to the women, potentially affecting the statistical power of some analyses. A replication analysis in a larger group is warranted. Moreover, the ApaI distribution significantly differs from the HWE, but this is consistent with an age-related positive selective pressure of the AA genotype as already described [14].

In conclusion, these results shed light on the complex relationship between *VDR* SNPs and aging, suggesting a role of ApaI on the “fragilization” of very old persons.

Although these are preliminary findings, our observations could be useful in designing ad hoc clinical studies on lifestyle, vitamin D supplementation, and rehabilitation treatments in aging.

## Figures and Tables

**Table 1:** Characteristics of study participants.

	Women (n = 127)	Men (n = 75)	*p*
Age	80.5 ± 15.4	79.7 ± 14.2	0.91
FI	0.27 (0.1–0.5)	0.23 (0.1–0.3)	**0.05**
25(OH)D μg/L	17.5 ± 15.1	20.9 ± 14.2	**0.04**
PTH ng/L	86.8 ± 68.7	61.5 ± 44.2	**0.002**
Calcium mg/dL	10.1 ± 0.9	10.2 ± 0.5	0.79
Posphorus mg/dL	3.7 ± 0.8	3.2 ± 0.5	**<0.001**

Data are expressed as mean ± standard deviation (SD) and/or median and interquartile range (IQR). FI: frailty index, PTH: parathyroid hormone. Statistically significant *p* values are in bold.

**Table 2:** Association between FI and *VDR* polymorphisms.

FI	Women (n = 50)			Men (n = 35)		
	B	*p*	95%CI	B	*p*	95%CI
TaqI	−0.02	0.56	−0.10 to 0.05	0.03	0.48	−0.06 to 0.12
BsmI	0.06	0.10	−0.01 to 0.14	−0.05	0.27	−0.15 to 0.04
ApaI	0.08	**0.03**	0.01 to 0.16	−0.05	0.37	−0.15 to 0.06
FokI	0.04	0.24	−0.03 to 0.12	0.06	0.13	−0.02 to 1.14

Multiple linear regression analyses between frailty index (FI) as dependent variable and each *VDR* polymorphism adjusted by age. TaqI (Tt + tt) vs. (TT), BsmI (Bb + bb) vs. (BB), ApaI (Aa + aa) vs. (AA), and FokI (Ff + ff) vs. FF. Statistically significant *p* values are in bold.

## Supplementary Materials

The following are available online at https://www.mdpi.com/2072-6643/12/9/2714/s1, Table S1: Genotype and allele distributions of polymorphisms of *vitamin D receptor*.
