# Supplementary material for: Vitamin D Receptor Polymorphisms in Sex-Frailty Paradox
Source: Nutrients. 2020 Sep 5;12(9):2714. doi: 10.3390/nu12092714 (PMC7551757; doi:10.3390/nu12092714)
Supplement: Supplementary file 1 [file nutrients-12-02714-s001.pdf]

Table S1. Genotype and allele distributions of polymorphisms *of Vitamin D receptor gene.*

VDR	**Women (n = 50)**	**%**	**Men (n = 35)**	**%**	**Chi Square**	***p***
**Rs73236 (TaqI)**						
**TT**	16	32.0%	13	37.1%		
**Tt**	26	52.0%	20	57.1%		
**tt**	8	16.0%	2	5.7%		
					2.11	0.35
**T**	58	58.0%	46	65.7%		
**t**	42	42.0%	24	34.3%		
					1.03	0.31
**rs1544410 (BsmI)**						
**BB**	15	30.0%	8	22.7%		
**Bb**	20	40.0%	18	51.4%		
**bb**	15	30.0%	9	25.7%		
					1.12	0.57
**B**	50	50.0%	34	48.6%		
**b**	50	50.0%	36	51.4%		
					0.03	0.85
**rs7975232 (ApaI) ***						
**AA**	16	32.0%	7	20.0%		
**Aa**	31	62.0%	23	65.7%		
**aa**	3	6.0%	5	14.3%		
					2.67	0.27
**A**	63	63.0%	37	52.9%		
**a**	37	37.0%	33	47.1%		
					1.74	0.18
**rs10735810 (FokI)**						
**FF**	22	44.0%	17	48.6%		
**Ff**	20	40.0%	14	40.0%		
**ff**	8	16.0%	4	11.4%		
					0.39	0.82
**F**	64	64.0%	48	68.6%		
**f**	36	36.0%	22	31.4%		
					0.38	0.53

Vitamin D receptor (VDR). All the genotype distributions are in Hardy Weinberg Equilibrium except for rs7975232 (ApaI) in women. * Chi square = 5.44 * $p = 0.02$.
